# Cost-effectiveness analysis of dasatinib versus imatinib in pediatric philadelphia chromosome-positive acute lymphoblastic leukemia patients in China

**DOI:** 10.1186/s12913-022-08971-7

**Published:** 2022-12-26

**Authors:** Wang Cao, Yuncui Yu, Yingpeng Qiu, Lu Liu, Hao Zhang, Liwei Shi, Yue Xiao, Lulu Jia, Ruidong Zhang, Xiaoling Wang

**Affiliations:** 1 Department of Pharmacy, Beijing Children’s Hospital, Capital Medical University, National Center for Children’s Health, Beijing, China; 2 Department of Clinical Research Center, Beijing Children’s Hospital, Capital Medical University, National Center for Children’s Health, Beijing, China; 3 National Health Development Research Center, National Health Commission, Beijing, China; 4 University of Sheffield, Sheffield, UK; 5 Department of Pharmacy, Qingdao Women and Children’s Hospital, Qingdao, China; 6 Hematology Center, Beijing Key Laboratory of Pediatric Hematology Oncology, National Key Discipline of Pediatrics (Capital Medical University), Key Laboratory of Major Diseases in Children, Ministry of Education, Beijing Children’s Hospital, Capital Medical University, National Center for Children’s Health, Beijing, China

**Keywords:** Philadelphia-positive acute lymphoblastic leukemia, Dasatinib, Imatinib, Cost-effectiveness analysis, Children

## Abstract

**Background:**

Dasatinib and imatinib are the recommended tyrosine kinase inhibitors (TKIs) for treating pediatric Philadelphia-positive acute lymphoblastic leukemia (Ph + ALL), and the one which has been approved indication in China is imatinib. Recently, clinical demand for Ph + ALL treatment is becoming unmet gradually with the increasing resistance of imatinib. There are some studies reporting the better efficacy and comparative safety of dasatinib compared with imatinib, but no economic comparison has been published. This study aims to supplement economic evidence by comparing the cost-effectiveness between imatinib and dasatinib in treating pediatric patients with Ph+ ALL in China, and to help clinical rational drug use via multi-dimensional value assessment.

**Methods:**

A decision tree model combined with a 10-year Markov model were established based on the disease progression. The parameters were collected from published literatures and our hospital’s electronic medical records. From the health system perspective, the incremental cost-effectiveness ratio (ICER) between the two treatment groups was calculated through cost-effectiveness analysis and then compared with the willingness-to-pay (WTP) threshold. The set WTP threshold in this study was 1 times per capita gross domestic product (GDP) of China, as recommended by the World Health Organization. Direct medical costs and quality-adjusted life years (QALYs) were calculated and discounted at 5%. The sensitivity analyses were conducted to assess the uncertainty and robustness of the results.

**Results:**

The total costs were CNY 1,020,995.35 and CNY 1,035,788.50 in imatinib group and dasatinib group during the 10-year simulation, and the total QALYs were 2.59 and 4.84. Compared with the imatinib treatment group, the ICER was around CNY 6,575.78/ QALY, which was less than the set threshold CNY 70,892/ QALY. The sensitive analyses indicated the robustness of the results.

**Conclusions:**

The cost-effectiveness analysis shows the potential cost-effective advantages of adding dasatinib comparing with adding imatinib for pediatric Ph + ALL patients in China under the set WTP threshold, which indicates that those patients could achieve more QALYs by paying acceptable fee.

## Background

Acute lymphocytic leukemia (ALL) is a rapidly progressing disease accounting for more than 70% of childhood leukemia. Philadelphia chromosome-positive acute lymphoblastic leukemia (Ph + ALL) is a relatively rare type of the disease, counting for 3% to 5%, characterized by an abnormal BCR-ABL1 fusion gene caused by the Philadelphia chromosome translocation [1]. The poor prognosis, high probability of recurrence, and serious economic and psychological burdens make Ph + ALL a critical illness in children [2]. Therefore, it is quite significant to find the effective and even cost-effective treatment to improve the clinical benefits for those pediatric patients.

Tyrosine kinase inhibitor (TKI) is a class of compounds which can inhibit the activity of tyrosine kinases. By inhibiting the phosphorylation of protein tyrosine residues, it can block the conduction of downstream signal pathways to inhibit the growth and metastasis of tumor cells [3]. Several studies have reported that the addition of TKIs to the original chemotherapy regimen could significantly improve the 5-year event-free survival (EFS) rate in Ph + ALL children by 20% to 30% [4–6]. Imatinib and dasatinib are the two representative TKIs in China. As the first-generation TKI, imatinib has been approved for treating pediatric Ph + ALL and also been involved in the list of medicines covered by the medical-insurance system in China. However, there are some shortcomings of imatinib in clinical practice, such as drug resistance. The better mechanism of dasatinib can help enhance the speed of curative effect and reduce drug resistance to meet the needs of rapid clinical disease control for patients [7]. Dasatinib could improve the complete response (CR) in induction therapy, and minimal residual disease (MRD) negative rate (< 0.01%) after induction therapy and before consolidation chemotherapy [8]. Several researches have indicated a better clinical efficacy and comparable safety of dasatinib compared with imatinib [8, 9]. The results of a head-to-head multi-center clinical trial in China assessing the efficacy of dasatinib and imatinib in pediatric Ph + ALL patients showed that, the 4-year EFS rate and overall survival (OS) rate could be significantly improved by 22.1% and 19.2% when using dasatinib, meanwhile the 4-year cumulative recurrence rate could be significantly reduced by 14.6% [9]. As the second-generation TKI, dasatinib has not been approved the indication for pediatric Ph + ALL by China National Medical Products Administration (NMPA), although it has been recommended by the diagnosis and treatment standard of childhood acute lymphoblastic leukemia (2018 version) [10]. Off-label use may bring some legal risks and huge economic burden, resulting in a dilemma for clinicians in selection of TKI.

More evidence about safety, efficacy and economy should be presented for facilitating indication approval and even for adjusting national drug policies. From the literature research, there had been a multi-center randomized controlled trial compared the EFS rate, OS rate, recurrence rate and adverse events between two drugs in target patients in China. But there were no relative studies about economic evaluation of the disease or economic comparison between the two drugs in treating the disease. Therefore, this study conducted a cost-effectiveness analysis to supplement economic evidence, based on the reported data of published literatures and real-world data from the electronic medical records (EMR) in our hospital.

## Method

### Patient population

In order to better reflect the characteristics of pediatric Ph + ALL patients and the disease progression, a retrospective real-world cohort survey was conducted to collect baseline characteristics, diagnosis and treatment information, cost information and so on. According to the pre-established inclusion and exclusion criteria, 32 cases of pediatric Ph + ALL patients were extracted from EMR system in our hospital, 14 of whom were treated with imatinib and the others were received dasatinib as the added TKI [9]. There were no significant differences in baseline characteristics between the two groups. The body surface area was assumed to be 0.8 square meter based on the median age of 7 years old in the retrospective cohort, referring to the growth standard. In the retrospective cohort study, the pediatric patients received approximately a 4-week induction therapy and a 44-week intensive chemotherapy without treatment interruption early or disease progression, and continuously used TKIs throughout the treatment period.

### Model structure

**Fig. 1:**
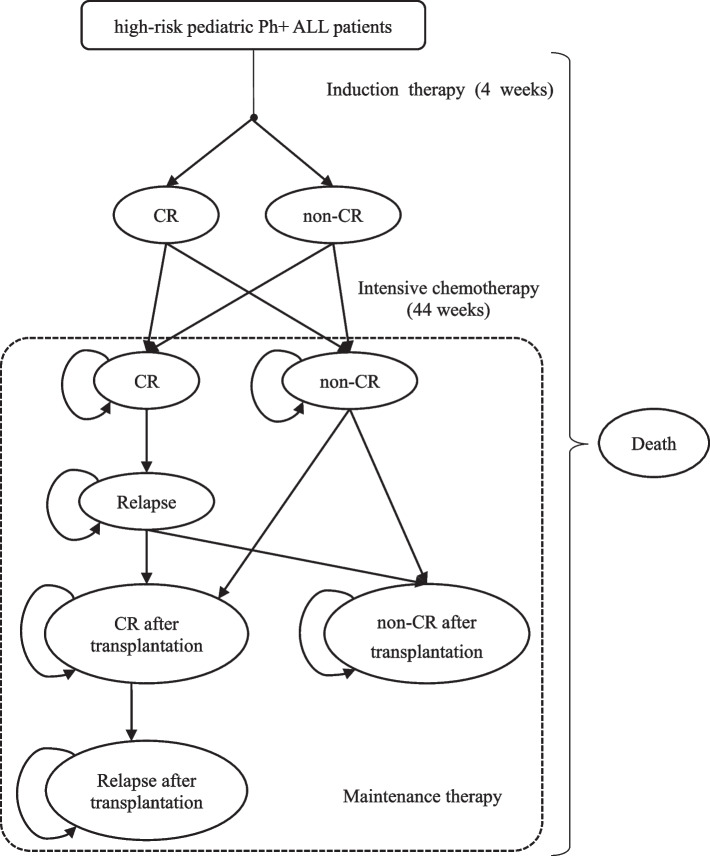
Simple Model Structure. CR complete response, non-CR non-complete response, HSCT hematopoietic stem cell transplantation

It was assumed that the patients were separated into two groups receiving either imatinib (300 mg/ day, per square meter of body surface area) or dasatinib (70 mg/ day, per square meter of body surface area) during the treatment period. According to the diagnosis and treatment standard of childhood ALL, the therapeutic pathways for pediatric Ph + ALL patients include induction therapy, intensive chemotherapy, and maintenance therapy. To better simulate the disease progression, a joint model of a decision tree and a 10-year Markov model was constructed, in which the disease progression was simplified to a few disease states, seen Fig. 1. The part of decision tree was used to simulate the progression in the induction therapy and intensive chemotherapy. At the end of the two period, patients would be in one of three states: (1) CR, (2) non-CR, and (3) death. After the intensive chemotherapy, patients would enter 10-years Markov simulation phase receiving maintenance therapy, with the initial state determined by the end of intensive chemotherapy. Those patients in non-CR state or relapsed state would receive hematopoietic stem cell transplantation (HSCT) if they have been assessed by clinicians, otherwise they might stay at the non-CR or relapse state and receive the chemotherapy again to keep alive. If transplantation fails, they might choose other possible therapeutic schedules, like immunotherapy. In each state, patients have the possibility to die. All patients would be in only one state in any one-year single cycle.

### Transition probabilities

In this study, the transition probabilities were from two sources. Parameters in the decision tree model were calculated based on the real-world retrospective cohort study. At the end of the 4-week induction chemotherapy, none of the patients treated with imatinib reached the CR state, while 3 patients treated with dasatinib (16.7%) became CR. At the end of the 44-week intensive chemotherapy, the CR rate for imatinib group and dasatinib group was 50% and 87.5% respectively [11]. For the Markov part, the parameters were collected from published articles and experts’ opinions. The annual probabilities from CR state to relapse or death state in maintenance therapy of the two group were estimated via transformation formula [12], based on the reported rate from the multi-center head-to-head clinical trial in China [9]. The transition parameters after transplantation were estimated referring to a relevant article [13]. Other parameters like the mortality of non-CR patients in maintenance therapy, the transplantation rate, and the success rate for transplantation were from experts’ opinions, seen Table 1.

**Table 1:** Base Case Parameter Values and Clinically Plausible Ranges for Model

Parameter	Base-case value (range)	Distribution	Reference
**Transition Probabilities**			
** Imatinib**			
non-CR after induction therapy	1	Beta	Cao 2021 [11]
non-CR after intensive chemotherapy	0.5	Beta	Cao 2021 [11]
Relapse in maintenance therapy	0.1000 (0.0415, 0.1729)	Beta	Shen 2020 [9]
Death of CR patients in maintenance therapy	0.0879 (0.0364, 0.1365)	Beta	Shen 2020 [9]
** Dasatinib**			
non-CR after induction therapy	0.833	Beta	Cao 2021 [11]
CR of CR patients in intensive chemotherapy	1	Beta	Cao 2021 [11]
CR of non-CR patients in intensive chemotherapy	0.875	Beta	Cao 2021 [11]
Relapse in maintenance therapy	0.0537 (0.0107, 0.1035)	Beta	Shen 2020 [9]
Death of CR patients in maintenance therapy	0.0304 (0.0099, 0.0504)	Beta	Shen 2020 [9]
** Death of non-CR patients in maintenance therapy**	0.6 (0.54, 0.66)	Beta	Experts' opinions
** Transplantation**			
Transplantation in non-CR patients	0.1 (0.09, 0.11)	Beta	Experts' opinions
Success in transplantation	0.4 (0.3, 0.5)	Beta	Experts' opinions
Relapse after transplantation	0.0582 (0.0354, 0.0806)	Beta	Lin 2019 [13]
Death in CR patients after transplantation	0.23 (0.21, 0.25)	Beta	Lin 2019 [13]
Death in non-CR/ relapse patients after transplantation	0.57 (0.49, 0.64)	Beta	Lin 2019 [13]
**Costs, CNY, per year**			
** Imatinib**			
Drug	104,857.2 (83,885.76, 125,828.64)	Gamma	Expense list
Other costs in induction therapy	71,019.18 (10,168.61, 170,406.88)	Gamma	Expense list
Other costs in intensive chemotherapy	266,871.23 (120,615.16, 398,733.09)	Gamma	Expense list
** Dasatinib**			
Drug	51,100 (40,880, 61,320)	Gamma	Expense list
Other costs in induction therapy	91,338.95 (26,639.48, 176,128.92)	Gamma	Expense list
Other costs in intensive chemotherapy	208,152.13 (141,714.97, 274,986.24)	Gamma	Expense list
** Other costs for maintenance in CR state**	57,500 (46,000, 69,000)	Gamma	Expense list
** Maintenance in non-CR state**	375,953.99 (244,489.6, 531,322.71)	Gamma	Expense list
** Transplantation**	250,000 (200,000, 300,000)	Gamma	Experts' opinions
** Maintenance in CR state after transplantation**	399,953.99 (268,489.6, 555,322.71)	Gamma	Expense list
** non-CR/ relapse after transplantation**	1,000,000 (800,000, 1,200,000)	Gamma	Experts' opinions
**Time horizon, year**			
** Imatinib**			
Induction therapy	0.0795	-	Cao 2021 [11]
Intensive chemotherapy	0.8164	-	Cao 2021 [11]
** Dasatinib**			
Induction therapy	0.0849	-	Cao 2021 [11]
Intensive chemotherapy	0.6822	-	Cao 2021 [11]
**Utilities, QALYs**			
** CR**	0.88 (0.82, 0.93)	Beta	Lin 2019 [13]
** non-CR/ relapse**	0.76 (0.7, 0.82)	Beta	Lin 2019 [13]
** CR after transplantation (first 5 years)**	0.8 (0.74, 0.86)	Beta	Lin 2019 [13]
** CR after transplantation (second 5 years)**	0.86 (0.8, 0.91)	Beta	Lin 2019 [13]
** non-CR/ relapse after transplantation**	0.73 (0.67, 0.79)	Beta	Lin 2019 [13]

*CR* Complete response, *non-CR* non-complete response, *QALY* Quality-adjusted life year

### Cost and utility

From the health system perspective, direct health care costs were collected and calculated, including the annual costs of TKIs, other medical-related treatment costs like inspection expenses and hospitalization expenses during induction therapy and intensive chemotherapy, the costs in CR or non-CR or relapse state during the maintenance period, and the cost of HSCT or potential treatment. In base case analysis, a 7-years-old child with a 0.8m^2^ body surface area was selected as a sample according to the characteristics of patients from retrospective study. The annual drug costs were calculated according to the usage and dosage specified in the instructions. Other cost parameters were derived from the expense lists from hospital's EMR or from experts’ opinions. As shown in Table 1, the costs were expressed in 2019 China yuan (CNY).

The outcome used in this study was quality-adjusted life years (QALYs). QALYs were calculated by multiplying the health utility of a specific health state by the number of years lived in that state. Utility score of different states were collected from published related literature [13], as presented in Table 1. It was assumed that non-CR state and relapse states have the same utility. The discount rate for cost and QALYs was 5% as recommended [14].

### Base case analysis

In base case analysis, total cost and total QALYs of the two group over a decade time horizon were calculated to estimate the incremental cost-effectiveness of dasatinib compared with imatinib through TreeAge Pro 2011 software. The incremental cost-effectiveness ratio (ICER) was defined as the differences in costs divided by the differences in health outcomes between two compared groups. The willingness to pay (WTP) threshold value for QALY was set at one capita of the gross domestic product (GDP) according to the 2020 China Guidelines for Pharmacoeconomic Evaluations [14]. As reported in China statistical Bulletin of National Economic and Social Development 2019, the 1 time GDP per capita is CNY 70,892 [15], which was the set threshold value.

### Sensitivity analysis

One-way sensitivity analysis was conducted to alter parameters including costs, transition probabilities and utilities in model input to assess the reliability and robustness of the findings. The upper and lower values for parameters reported in the data sources are preferred. If the variance or the range were not reported in references, the fluctuation amplitude of cost and utility parameters would be ± 20% of the baseline value, and ± 10% for transition probabilities according to experts’ consultation. The annual discount rate fluctuated between 3 and 8%.

A probability sensitivity analysis was also conducted through Monte Carlo simulation to explored the correlation between the uncertainty of input parameters and model outcomes. The input parameters were randomly drawn from the assigned parametric distributions in one thousand Monte-Carlo simulation, where costs were assumed to obey a Gamma distribution together with utility and transition probability a Beta one.

The original drug price was used to calculate the annual drug costs of the two TKIs in the base case analysis. Considering lower price of generic drugs may have an impact on the cost-effectiveness outcomes, a scenario analysis was necessary to be conducted to calculate the ICER under the price of marketed generic drugs with other conditions remain unchanged.

## Results

### Base case results

The base case analysis showed that total QALYs for patients under dasatinib or imatinib treatment were 4.84 and 2.59 respectively, implying the increment of 2.25 QALYs during the 10-years simulative time. Meanwhile, the total cost of patients treated with dasatinib was CNY 1,035,788.50, with an increase of CNY 14,793.15 than those treated with imatinib, shown in Table 2. Compared with adding imatinib as the treatment, ICER of adding dasatinib was about CNY 6,575.78/QALY, which was less than the WTP threshold, indicating the cost-effective advantage.

**Table 2:** Base case cost-effectiveness analysis results

Strategy	Total		Incremental		ICER
	Cost (CNY)	QALYs	Cost (CNY)	QALYs	
Imatinib	1,020,995.35	2.59	-	-	-
Dasatinib	1,035,788.50	4.84	14,793.15	2.25	6,575.78

*QALY* Quality-adjusted life year, *ICER* Incremental cost effectiveness ratio

### Sensitivity analysis results

**Fig. 2:**
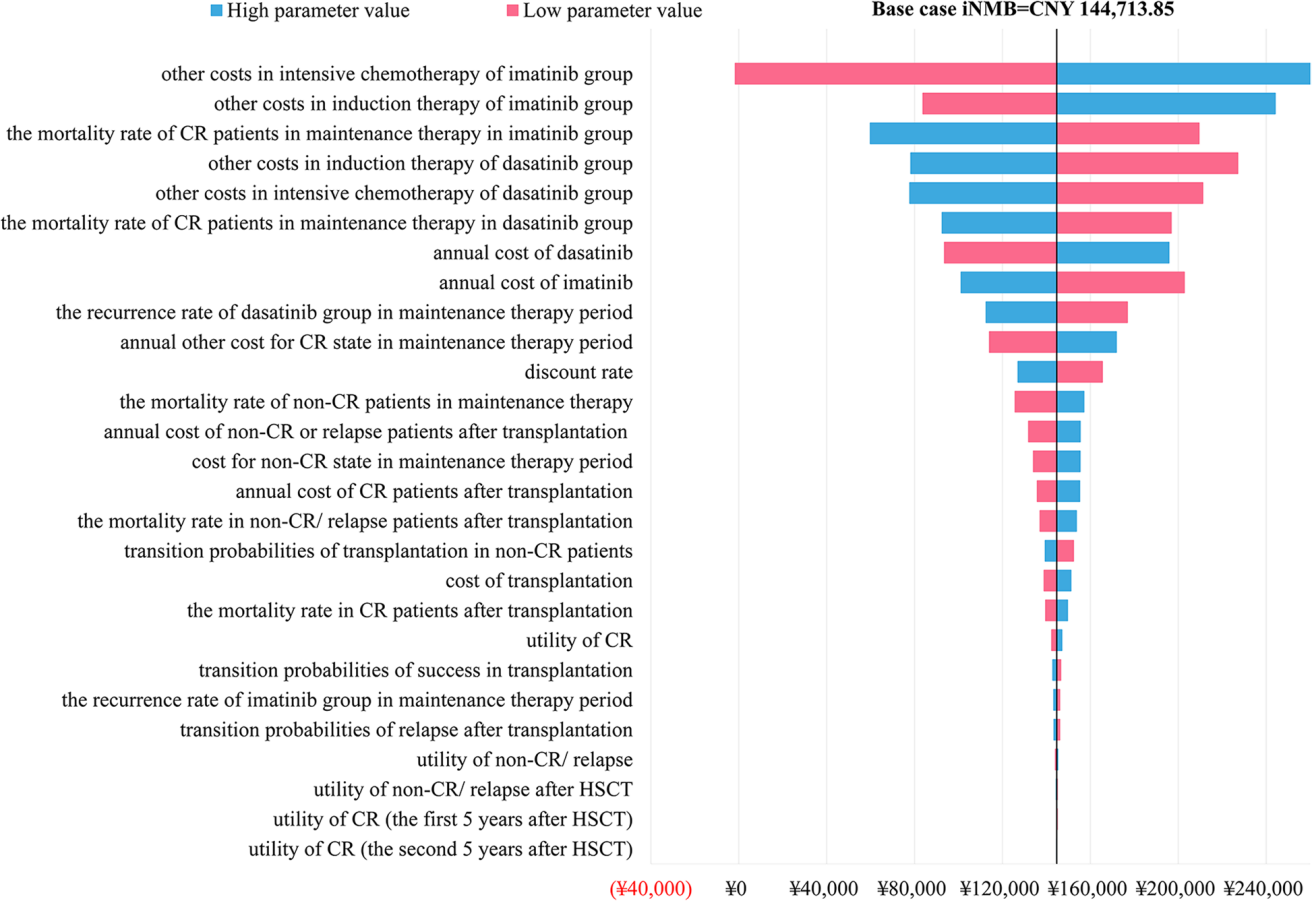
One-way sensitivity analysis (Tornado diagram of the incremental net monetary benefit)

**Fig. 3:**
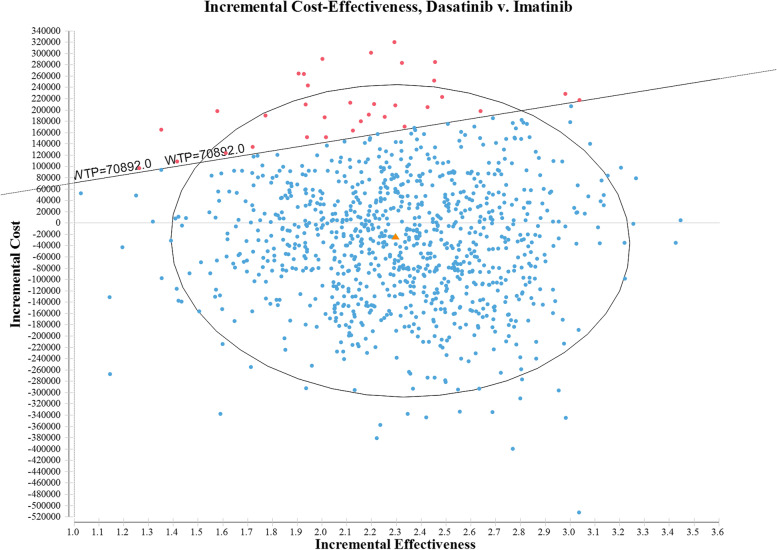
Scatter plot of ICER, dasatinib vs. imatinib. Pink dots indicate that imatinib has the cost-effectiveness advantage; blue dots: dasatinib has a cost-effectiveness advantage; orange triangle: the datapoint reflecting mean incremental QALYs (2.298590184 QALYs) and mean incremental costs (CNY -26,311.12355)

**Fig. 4:**
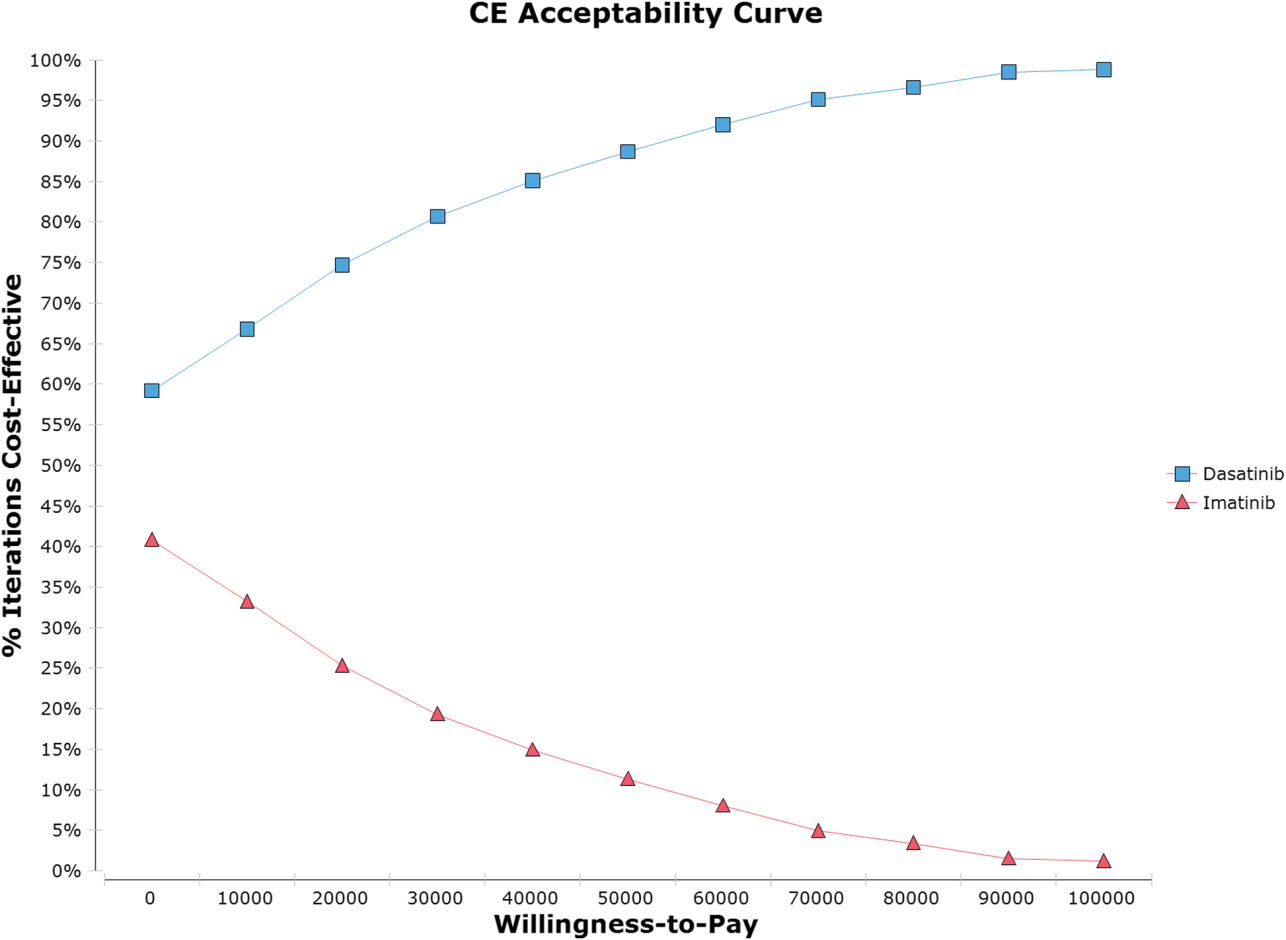
Cost-effectiveness acceptable curve

A series of one-way sensitivity analysis proved the robustness of the results. As presented in Fig. 2, the incremental net monetary benefit (iNMB) of base case was CNY 144,713.85. The horizontal line showed how iNMB changes with parameter fluctuations. The first five factors that greatly influenced iNMB including other costs in intensive chemotherapy of imatinib group (except imatinib costs), other costs in induction therapy of imatinib group (except imatinib costs), the mortality rate of CR patients in maintenance therapy in imatinib group, other costs in induction therapy of dasatinib group (except dasatinib costs) and other costs in intensive chemotherapy of dasatinib group (except dasatinib costs). According to the scatter plot of ICER between two groups (Fig. 3), there was a 96.7% probability of dasatinib being more cost-effective under the set WTP threshold. With the increasing of WTP, the acceptability of adding dasatinib as the treated TKI was greatly improved, which was obviously shown in Fig. 4. Under the scenario analysis and assumption, the annual costs for generic dasatinib and imatinib were CNY 28,207.2 and CNY 8,555.6 respectively, resulting in an increase of ICER from CNY 6,575.78/QALY to CNY 58,887.82/QALY, which was still lower than the set threshold.

## Discussion

Understanding the value of a new or alternative clinical intervention is crucial to guide rational clinical use of drugs. As the second generation TKI, dasatinib can help pediatric Ph + ALL patients control the disease progression more quickly and improve the survival rate. Considering the higher cost of the full treatment course, it is necessary to take the economic evidence into consideration when evaluating the value of adding dasatinib in treatment. After literature research, there were no published economic evaluation studies comparing dasatinib and imatinib in treating pediatric Ph + ALL so far, but there are some about treating chronic myeloid leukemia. Those studies presented different economic findings under different research background [16–21]. Therefore, it is necessary to conduct pharmacoeconomic evaluation research for specific diseases in specific countries. This study may help fill the research gap to some extent.

To the best of our knowledge, our study is the first to establish a combined economic model based on real-world data to evaluate the cost-effectiveness of dasatinib compared with imatinib in treating pediatric Ph + ALL patients in China. Although the annual cost of original dasatinib is cheaper than that of imatinib, shown in Table 1, there would be other costs during the whole treatment period like the costs of chemotherapy drugs, hospitalization, etc. To better reflect the costs in clinical treatment, expense list was extracted from the EMR system of our hospital for calculation. The results showed that the incremental cost of adding dasatinib than adding imatinib during the simulation period was CNY 14,793.15, but with an incremental effectiveness of 2.25 QALYs. It implied that adding dasatinib was a cost-effective and acceptable choice when comparing the ICER with the set WTP threshold, both in base case and sensitive analyses. In addition, considering that using dasatinib could reduce the treatment costs due to relapse by reducing recurrence rate [9, 22], we compared this potential saving cost with the cost of dasatinib over a year. The result indicated that the combination of dasatinib is a cost-saving treatment, which could save about CNY 34,974.57 (CNY 51,100 vs. CNY 86,074.59).

In this study, the parameters were mainly derived from real-world retrospective study and the head-to-head clinical trial. The baseline characteristics of the patients enrolled from the EMR data of our hospital were similar to those enrolled in the clinical trial [8]. Therefore, the assumption of basic characteristics of patient sample based on real-world data was acceptable. What’s more, the safety and efficacy results of our retrospective study were consistent with those reported in the trial.

It should be pointed out that, although the WTP threshold was set according to the China Guidelines for Pharmacoeconomic Evaluations guideline in the study, there has been no established standard for the value of QALY in China yet. Some experts consider that the more appropriate threshold would be 63% of GDP per capita [23], which means the current threshold used in China is much too high. If taking this into account, the threshold would be decreased to CNY 44,661.96/QALY, which might lead to changes of economic evaluation results. Nowadays, both the original drugs and generic drugs are used in clinical treatment. The new ICER of original drugs is still less than the new threshold (CNY 6,575.78/QALY vs. CNY 44,661.96/QALY), while the ICER of the generic drug would be higher (CNY 58,887.82/QALY vs. CNY 44,661.96/QALY). Taking this into account, the annual cost of dasatinib should be reduced to CNY 21,928.36 to make it an economical choice than generic imatinib, which means the unit price should be reduced from CNY 69 per piece to CNY 53.64.

There are also some limitations in this study. Firstly, we simplified the disease treatment pathway into three periods based on the experts’ opinion, which may not fully reflect the complex progression in real situations. Secondly, the transition probabilities in decision tree were calculated based on real-world data from one single hospital, while the small sample size of patients may not represent other regions. Multicentric real-world data can be collected in the next step to enhance the reliability of the results. Thirdly, the transition probabilities in Markov model were estimated from a 4-year OS rate and EFS rate, which may overestimate or underestimate the probability of metastasis of actual disease progression. Although we have incorporated significant sensitivity analyses to address these limitations and find our results to be robust, it is necessary to conduct further clinical trials or long-term follow-up visiting for updating the parameters and results, thus providing more evidence for using dasatinib.

## Conclusion

This study is the first cost-effectiveness analysis comparing the economic advantages between dasatinib and imatinib for pediatric Ph + ALL patients in China. Our results suggest that using dasatinib as the added TKI might be a cost-effective choice under the health system perspective since the acceptable increasing cost would bring with more efficacy, which may help promote rational clinical use and improve the quality of life of those patients.

## Data Availability

The datasets generated or analyzed during the current study are from the following published article: (1) Shen S, Chen X, Cai J, et al. Effect of Dasatinib vs Imatinib in the Treatment of Pediatric Philadelphia Chromosome-Positive Acute Lymphoblastic Leukemia: A Randomized Clinical Trial. JAMA Onco. 2020;6(3):358–366, doi: 10.1001/jamaoncol.2019.5868., and (2) Cao W, Yu Y-C, Liu L, et al. Comprehensive Clinical Evaluation of Dasatinib in the Treatment of Children with Philadelphia-positive Acute Lymphoblastic Leukemia. Chinese Journal of Drug Evaluation. 2021;38(03):183–190, doi:10.3969/j.issn.2095-3593.2021.03.002.

## Abbreviations

CR: Complete response
EFS: Event-free survival
EMR: Electronic medical records
GDP: Gross domestic product
HSCT: Hematopoietic stem cell transplantation
ICER: Incremental cost-effectiveness ratio
iNMB: Incremental net monetary benefit
MRD: Minimal residual disease
NMPA: National Medical Products Administration
OS: Overall survival
Ph + ALL: Philadelphia-positive acute lymphoblastic leukemia
QALYs: Quality-adjusted life years
TKI: Tyrosine kinase inhibitors
WTP: Willingness-to-pay
